# Climate anomalies and competition reduce establishment success during island colonization

**DOI:** 10.1002/ece3.9402

**Published:** 2022-10-08

**Authors:** Daniel J. Nicholson, Robert J. Knell, Rachel S. McCrea, Lauren K. Neel, John David Curlis, Claire E. Williams, Albert K. Chung, William Owen McMillan, Trenton W. J. Garner, Christian L. Cox, Michael L. Logan

**Affiliations:** ^1^ Queen Mary University of London London UK; ^2^ Smithsonian Tropical Research Institute Panama City Panama; ^3^ Zoological Society of London London UK; ^4^ University of Texas at Arlington Arlington Texas USA; ^5^ Lancaster University Lancaster UK; ^6^ Arizona State University Tempe Arizona USA; ^7^ University of Michigan Ann Arbor Michigan USA; ^8^ University of Nevada Reno Nevada USA; ^9^ Princeton University Princeton New Jersey USA; ^10^ Florida International University Miami South Florida USA

**Keywords:** *Anolis*, climate change, community ecology, population dynamics, species interactions

## Abstract

Understanding the factors that facilitate or constrain establishment of populations in novel environments is crucial for conservation biology and the study of adaptive radiation. Important questions include: (1) Does the timing of colonization relative to stochastic events, such as climatic perturbations, impact the probability of successful establishment? (2) To what extent does community context (e.g., the presence of competitors) change the probability of establishment? (3) How do sources of intrapopulation variance, such as sex differences, affect success at an individual level during the process of establishment? Answers to these questions are rarely pursued in a field‐experimental context or on the same time scales (months to years) as the processes of colonization and establishment. We introduced slender anole lizards (*Anolis apletophallus*) to eight islands in the Panama Canal and tracked them over multiple generations to investigate the factors that mediate establishment success. All islands were warmer than the mainland (ancestral) environment, and some islands had a native competitor. We transplanted half of these populations only 4 months before the onset of a severe regional drought and the other half 2 years (two generations) before the drought. We found that successful establishment depended on both the intensity of interspecific competition and the timing of colonization relative to the drought. The islands that were colonized shortly before the drought went functionally extinct by the second generation, and regardless of time before the drought, the populations on islands with interspecific competition declined continuously over the study period. Furthermore, the effect of the competitor interacted with sex, with males suffering, and females benefitting, from the presence of a native competitor. Our results reveal that community context and the timing of colonization relative to climactic events can combine to determine establishment success and that these factors can generate opposite effects on males and females.

## Graphical abstract

Our results reveal that community context and the timing of colonization relative to climatic events can combine to determine establishment success.

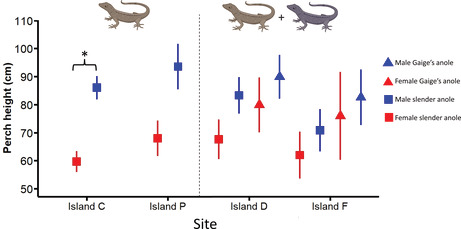

## 1 INTRODUCTION

Since the beginning of life on earth, organisms have expanded their ranges and colonized novel environments (Whittaker, 2007). In the Anthropocene, the number of these colonization events has dramatically increased due to human activity, and alien species can cause problems if they become established and begin to spread (Blackburn et al., 2011; Capellini et al., 2015; Hoskin, 2011; Savidge, 1984; Wan et al., 2019). We define “establishment” as the persistence of an alien population for several generations after colonization such that it becomes a viable member of the local community. Successful establishment is a consequence of multiple complex processes, such as the community structure and climate of the region, as well as genetic, phenotypic, and demographic features of both the alien species and members of the native community (Dyer et al., 2017; Redding et al., 2019). Few studies have examined the respective roles of these factors (or their interactions) on the same time scale over which colonization and establishment occur (weeks to years, depending on the species; Alzate et al., 2020; Ezard et al., 2009). Understanding the phenomena that limit or enhance the establishment of alien species in new regions is a crucial step if we are to predict and therefore mitigate biological invasions.

The ability to reach an environment (henceforth, “colonization”) does not guarantee establishment success (Blackburn et al., 2011; Losos & Spiller, 1999). After colonization, populations are likely to be small in size due to a limited number of initial arrivals and a sudden increase in selection intensity in the new environment (Calsbeek & Cox, 2010; Endler, 1986). If the population does not immediately go extinct, selection or plasticity (or both) may drive the mean phenotype toward the local fitness optimum, and the population should begin to recover (West‐Eberhard, 2003). However, if a species arrives in a new environment in close temporal proximity to a chance climate event (e.g., a heat wave, drought), population size may be reduced below the recovery threshold before the species is able to grow in and adapt to the new environment. If, however, a population has had several generations to grow and adapt to the local environmental conditions before the onset of the climate event, then it might be able to recover (Chevin et al., 2010; Wang & Althoff, 2019). Climate anomalies may also increase the probability that a population establishes if the anomaly results in a local environment that better matches the ancestral range or reduces the effects of antagonistic interactions. Regardless, these dynamics may become increasingly important in the Anthropocene, as climate change is predicted to cause an increase in the frequency of extreme weather events (IPCC, 2021).

In addition to novel climates, alien species will encounter communities of competitors, predators, and parasites that differ from their ancestral range. Interspecific competition is a key mediator of invasion dynamics in many systems and often has strong effects on the fitness of both the invader and members of the native community (Alzate et al., 2017; Case et al., 1994; Petren & Case, 1996). Competitive interactions during invasion can involve exploitation competition, where different species negatively affect each other via the consumption of a common resource without direct interaction (Corlatti et al., 2019; Högstedt, 1980), and interference competition, where individuals of different species interact directly as they fight over access to resources (Corlatti et al., 2019; Grether et al., 2009). Regardless of the type of interspecific competition that occurs, these interactions are often asymmetrical, with one or more members of the interaction suffering more than others. For example, Kolbe et al. (2016) found that recently introduced crested anoles (*Anolis cristatellus*) were four times more abundant and perched lower in vegetation at sites without the previously introduced and firmly established brown anoles (*Anolis sagrei*) in urban environments in Miami. Even in situations where an alien species colonizes an environment that is more suitable than its original habitat, interspecific competition can reduce the survival and fitness of the invader (Cox et al., 2020; Petren & Case, 1996). The community context, and in particular the competitive landscape, in which a colonizing population finds itself is likely to play an important role in mediating establishment success.

In addition to exogenous factors such as climate and competition, endogenous features of the colonizing population such as trait differences between males and females may also affect establishment success. Males and females in many species differ in a wide range of traits, and these phenotypic differences might interact with aspects of the local environment to influence the sex ratio of the alien population and its probability of establishment (Fargevieille et al., 2020; Iglesias‐Carrasco et al., 2020). Females generally must disproportionately invest resources directly into reproduction, whereas males tend to spend more energy on competition (Agrawal, 2001; Iglesias‐Carrasco et al., 2020). Thus, when a colonizing population disperses to an environment with a high intensity of interspecific competition, it is possible that males may be at a disadvantage compared to females (Iglesias‐Carrasco et al., 2020; Nicolaus et al., 2009). Moreover, differences in the ecology and physiology of males and females might impact their survival with respect to local climate and exposure to extreme weather events (Gianuca et al., 2019). Some species of *Anolis* lizards, for example, exhibit differential habitat use between the sexes, which translates to sex‐based differences in diet and thermal tolerance (Logan et al., 2021; Losos, 2009; Rosso et al., 2020). This variation among the sexes in important phenotypes may translate to sex biases in fitness during the process of invasion.

We investigated how environmental circumstances, namely, community context and the timing of an extreme environmental perturbation relative to colonization, influenced establishment success in populations of the Panamanian slender anole lizard (*Anolis apletophallus*, henceforth, “slender anole”) that we translocated to eight islands (henceforth, “experimental islands”) in the Panama Canal. Slender anoles are a good model for understanding the dynamics of establishment as they are small, abundant, and have a short generation time with nearly 100% annual population turnover (Andrews, 1991; Andrews & Nichols, 1990). We introduced half of these populations in 2017 during a normal climatic year and half in 2018 in the months before a drought hit central Panama during the dry season (December 2018 – May 2019). This drought resulted in half of the dry season rainfall compared to the previous year and was so severe that it caused authorities to limit the amount of cargo that ships could bring through the Panama Canal (Fountain, 2019). Even in nondrought years, the experimental islands were warmer than the mainland (ancestral) environment, and two of the islands (henceforth, “two‐species islands”) had a native anole species *Anolis gaigei* (henceforth, “Gaige's anole”), which is a competitor of the slender anole. Despite an unbalanced design whereby fewer than half of the islands had a native competitor and both two‐species islands received slender anoles in the same year, we nevertheless took advantage of the unanticipated drought to investigate how interspecific competition, climate stochasticity, and the timing of establishment interact to affect the chances of establishment and invasion success. We hypothesized that (1) the populations that had several generations to adapt to local conditions would be at higher densities before the onset of the drought and would therefore be more likely to establish, (2) the presence of a native competitor would reduce the establishment success of slender anoles irrespective of the timing of colonization, and (3) establishment success of males and females would differ (i.e., unequal sex ratios of colonizing populations) because of ecological and physiological differences between the sexes.

## 2 METHODS

### 2.1 Study sites

We studied colonization and establishment dynamics in populations of a recently introduced species using an experimental island system in Panama's Lake Gatún. Lake Gatún is a 425 km^2^ artificial lake created by the damming of the Chagres River during the construction of the Panama Canal in 1913 (Giles Leigh Jr et al., 1993). We used eight small islands (areas ranged from 802 to 6210 m^2^, mean = 3223 m^2^) that were formerly hilltops before the valley was flooded. The islands in Lake Gatún are well within the natural range of slender anoles and this species almost certainly occupied the Chagres Valley before it was flooded (the larger islands in the lake have resident populations to this day). Thus, transplanting lizards around this area is not of ethical concern in that they are being moved relatively short distances within their natural range and to small, isolated plots of land that play a minimal role in the ecology of the broader region. We thoroughly surveyed each of these islands before transplantation to ensure that they did not have resident populations of slender anoles. Two of the islands (Islands D and F) had a different, resident species of anole called Gaige's anole (*Anolis gaigei*). Nevertheless, all eight experimental islands had much lower competitor diversity (slender anoles coexist with at least seven other anole species on the adjacent mainland), and they probably had lower predator and parasite diversity compared to the mainland due to their small size and isolation. This reduced biological complexity on the experimental islands and simplified the number of variables we needed to consider as potentially affecting establishment success (Arnold & Asquith, 2002; Giles Leigh Jr et al., 1993; MacArthur & Wilson, 1967).

### 2.2 Collection of founding individuals

The founder generation of slender anoles consisted of 560 lizards that we captured from a single site in Soberanía National Park on mainland Panama near the town of Gamboa (9°08′00.1″N, 79°43′11.0″W). We transplanted lizards to four islands (70 individuals per island, equal sex ratios) in 2017 (between July and September). We then repeated this process for four new islands in 2018 (between July and September). Thus, our data set includes eight islands that were “colonized” over a two‐year period. We caught adult lizards (>38 mm snout‐vent‐length, or “SVL”) either by hand or with a lizard catch‐pole (fishing rod and line with a slipknot). Lizards were transported to the Smithsonian facility in Gamboa for morphological, physiological, and genetic sampling procedures that were associated with other projects (Cox et al., 2020; Logan et al., 2021; Neel et al., 2021). In captivity, lizards were housed in small plastic terraria for a maximum of 48 h. We included a balled‐up piece of paper towel saturated with water as a source of humidity within each terrarium. Due to the short processing time, we did not feed captive individuals.

### 2.3 Transplantation to islands and mark‐recapture

Before transplanting lizards to experimental islands, we implanted visual elastomers (VIE codes; Northwest Marine Technology, Inc.) to give each individual a unique and reliable identifier (Daniel et al., 2006; Nicholson et al., 2015). Lizards were then randomly assigned to islands and released in batches (20–40 lizards per batch). We conducted mark‐recapture surveys on the 2017 founder (F_0_) populations between October and December 2017, on their adult offspring (F_1_ generation) between June and November 2018, and on the third generation (F_2_) between June and September 2019. We conducted mark‐recapture surveys on populations we transplanted in 2018 on their first‐generation adult offspring (F_1_) between June and September 2019. During these recapture surveys, we systematically searched each island approximately twice per week. To conduct these searches, we divided an island into nonoverlapping “lanes” that circled the island. One observer would occupy each lane and slowly move around the island until they encountered a lizard. When we encountered a new lizard (adult recruit) on an island, we first measured that lizard's perch height and diameter using a tape measure and digital calipers, respectively. These individuals were then taken to the Smithsonian Facility in Gamboa for additional phenotypic measurements and tagging, after which they were released back onto their island of origin and spot of capture within 48 h. Any time we recaptured a lizard, we again recorded its perch height and diameter along with its VIE code, but we then immediately released it at the spot of capture. The same methods (for both initial captures and recaptures) were replicated for Gaige's anoles when caught on the islands in which they occur.

We analyzed habitat use between species and sex across islands by fitting a linear mixed‐effects model, with “lizard ID” as a random effect to account for repeated measures. Perch height was the dependent variable with “island,” “sex,” and “species” included as fixed factors. Perch height was log_10_ transformed to meet the model assumption for normally distributed residuals.

### 2.4 Local and regional climate

We monitored regional temperature and precipitation in the Lake Gatun area using the El Claro weather station on Barro Colorado Island (BCI: 9.1521°N, 79.8465°W). While we did not transplant lizards to BCI, this site is close by and centrally located to our experimental islands. The El Claro weather station records total daily rainfall (mm) and temperature (°C) every 15 min.

Our methodology for measuring local environmental temperature distributions for slender anoles has been reported in detail elsewhere (Cox et al., 2020; Logan et al., 2021; Neel et al., 2021). In brief, we coated iButton temperature loggers (calibrated at factory: Embedded Data Systems, Lawrenceburg, KY, USA) in PlastiDip (PlastiDip International, Blaine, MN, USA) for waterproofing and then glued them to a short piece of wooden trim. We deployed these data loggers on each of our islands, where they recorded environmental temperatures every 120 min (we staggered start times so that multiple loggers recorded temperatures within every hour interval of the study period) between July 2017 and September 2019. We deployed temperature data loggers randomly in space across all eight islands (mean of 23 data loggers per island for most years but see caveat about Island D in 2019, below). We used random cardinal directions and distances (0–5 m in 1 m increments) from haphazardly chosen points covering as much of each island as possible and then strapped the data loggers to branches using zip ties at random heights (0.5–2 m in 0.5 m increments) and orientations (above, to the side, or below the branch; Logan et al., 2021).

We used linear models to compare weekly rainfall among years, with separate models for wet and dry seasons. We used weekly rainfall data to avoid zero inflation caused by many rainless days. All analyses (here and below) were conducted in R version 3.5.3 (R Core Team, 2019). We used mixed‐effect models to compare both daily regional temperatures (BCI weather station) and local island temperatures (OTM data) across years and seasons. We used “Date” as a random effect to account for repeated daily measures. Mixed‐effect models were implemented in the *lme4* package in R (Bates et al., 2015). Diagnostic plots were checked for appropriate residual distributions for all fitted models.

### 2.5 Population growth and survival models

To calculate changes in population size and individual survival probabilities over time, we used open versions of the Robust Design model (Pollock, 1982) created in the *RMark* package (Laake, 2013). For both survival and population size models, we classed each year (breeding season) as the primary capture occasion and weekly captures within each season as the secondary occasions, of which there were 14 in 2017 and 13 in both 2018 and 2019. We constrained the emigration and immigration (γ″, γ′) parameters to zero under the reasonable assumption that rates of emigration and immigration were negligible in an island system. To calculate survival probability separately for males and females we included “sex” as a grouping factor. Models were ranked using Akaike's Information Criterion corrected for small sample size (AICc), and the final models were substantially lower in value than the next lowest value (Burnham & Anderson, 2002; Table S1). It was not possible to use the Robust Design Model for evaluating population sizes and survival for the islands introduced in 2018, as we did not conduct mark‐recapture surveys on these islands immediately after introduction in 2018, and there were virtually no remaining individuals on any of these islands in 2019. Thus, population size estimates for 2019 represent the only remaining survivors after 2 months of extensive searching. We also estimated slender anole population density on each island by dividing our population size estimate by the total area of each island as calculated using Google Earth images in ImageJ v.1.52a (Schneider et al., 2012).

## 3 RESULTS

### 3.1 Climate anomalies and establishment success

**FIGURE 1:**
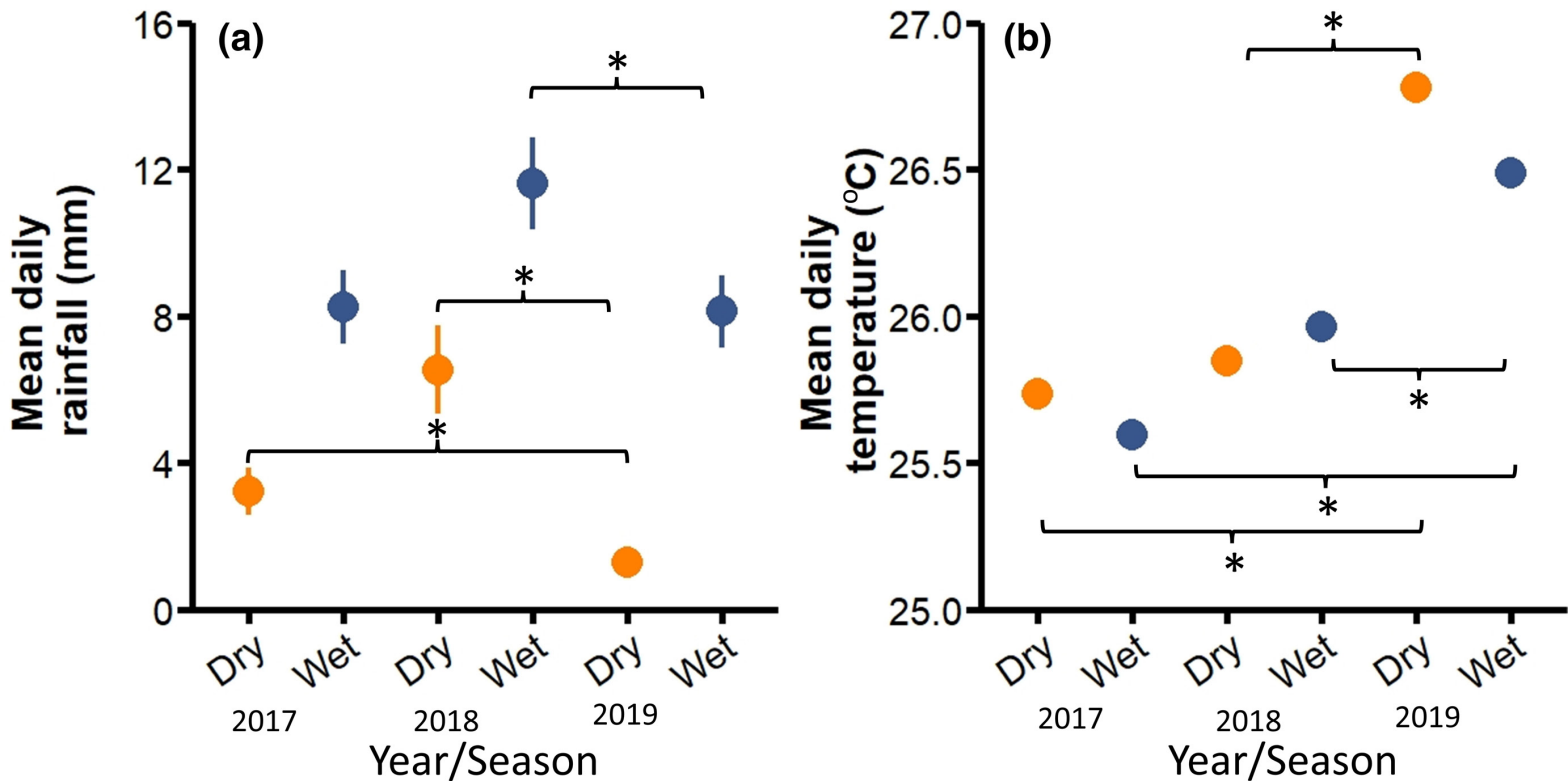
Differences between mean weekly rainfall (a) and mean weekly temperature (b) for the wet (blue) and dry (orange) seasons, from 2017 to 2019. Rainfall and temperature were recorded from the El Claro weather station on Barro Colorado Island. Symbols represent mean ± *SEM* (some *SEM* are too small to be visible). Brackets with asterisks denote comparisons that were significantly different.

Central Panama experienced a major drought from December 2018 to May 2019 with almost no rain recorded for four straight months (Paton, 2019). According to precipitation data recorded at the El Claro weather station on Barro Colorado Island from 1929 onwards, the 2019 dry season was the most severe dry season in more than 30 years, the seventh driest in almost a century, and was 65% drier than the average dry season. Within the timeframe of our study, rainfall was significantly lower in the dry season of 2019 compared to the previous two dry seasons (*F*_(2,61)_ = 3.633, *p =* .032; Figure 1a). Rainfall was also significantly higher in the 2018 wet season during the second introduction period compared to 2017 and 2019 (*F*_(2,61)_ = 3.656, *p =* .029; Figure 1a). Temperature data from the El Claro weather station on BCI showed that 2019 was also significantly hotter than the previous 2 years (as confirmed by the coefficient estimates and standard errors in a mixed‐effects model; Table S2; Figure 1b). This regional scale temperature difference was reflected at local scales, as well. Data from our environmental temperature data loggers showed that all islands were warmer during the 2019 drought period than during the same period in the year before (Island C: *t*_(48)_ = −5.52, *p* = <.001, temperature difference = 0.4°C; Island F: *t*_(39)_ = −12.07, *p* = <.001, temperature difference = 0.9°C; Island P: *t*_(42)_ = −8.36, *p* = <.001, temperature difference = 0.5°C). The difference was nonsignificant on Island D, but this was almost certainly a result of equipment failure which vastly reduced sample size (*N* = 2) in 2019. The timing of this drought meant that it primarily affected the F_1_ generations of the islands that were founded in 2017 and the F_0_ generations of the islands that were founded in 2018.

**FIGURE 2:**
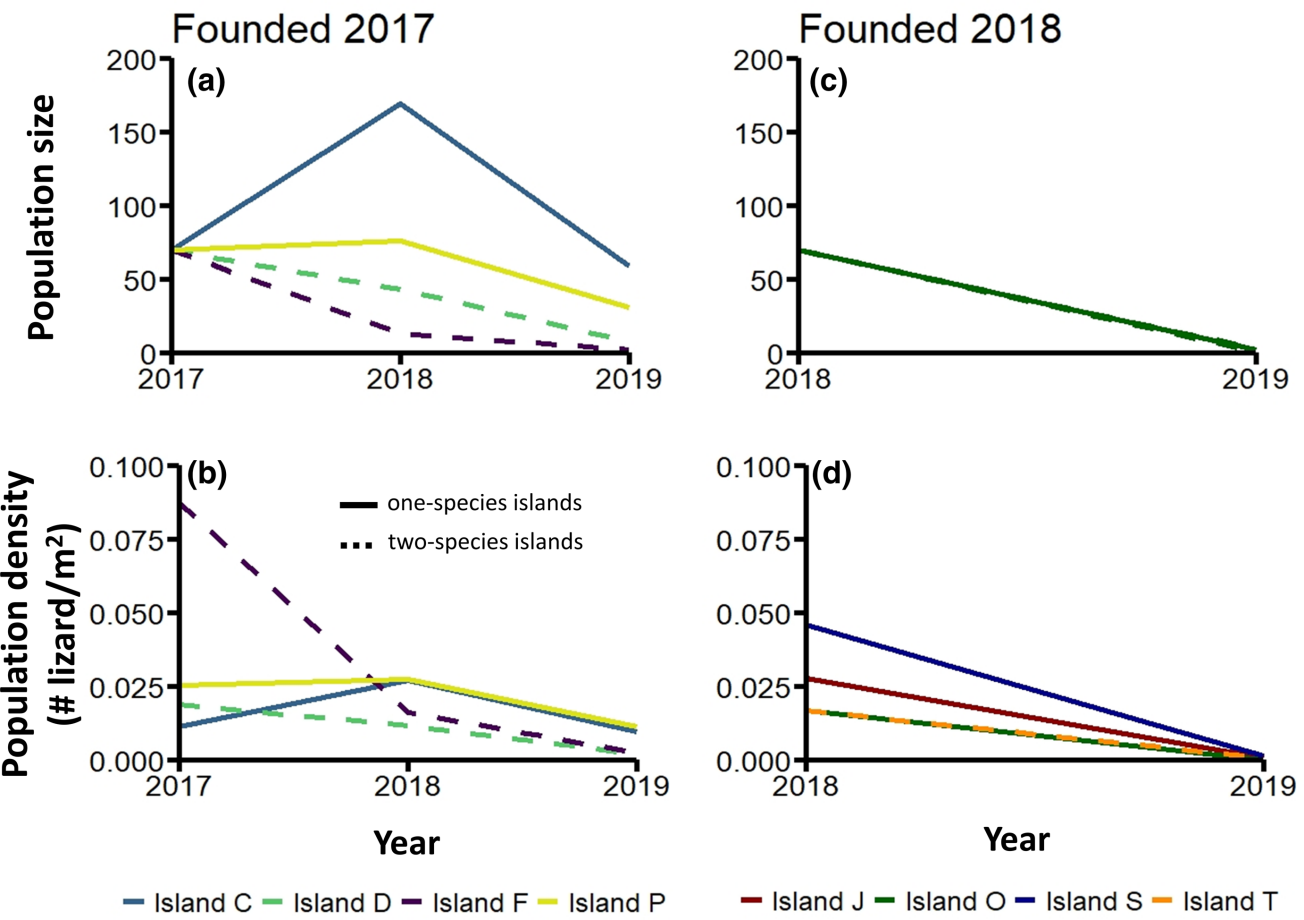
Changes in the size and density of slender anole populations that “colonized” islands with (dashed lines) or without (solid lines) a native competitor and either two generations before (left column), or within a few months of (right column), the onset of a severe drought. While slender anole population size (a) and density (b) either increased or remained stable on one‐species islands that were colonized two generations before the drought, they declined on the two‐species islands over the length of the study. Even though all the populations we translocated in 2018 about 4 months before the onset of the drought were placed on one‐species islands, both population size (c) and density (d) crashed within one generation. Note that lines for several islands completely overlap on panels c and d.

After the drought, slender anole population size declined from 2018 to 2019 across all eight experimental islands (credible intervals for population sizes between these years were entirely or mostly non‐overlapping for all islands; Table S3), including islands that were colonized in 2017, which had previously experienced population growth (Figure 2a–d). The islands in 2018 that were founded only 4 months before the start of the drought experienced the steepest rates of decline, with all populations reduced to four or fewer individuals in 2019 (Figure 2b,d).

### 3.2 Competition and establishment success

**FIGURE 3:**
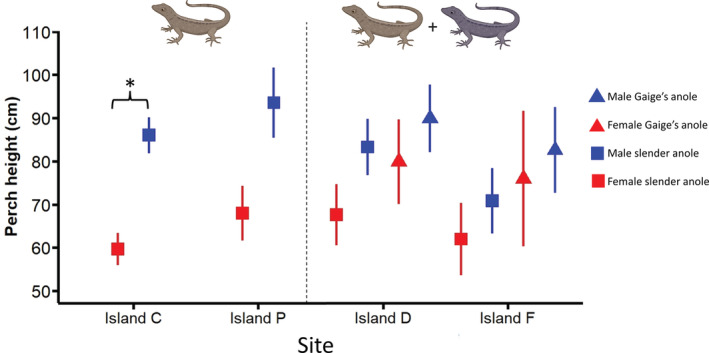
Habitat use (perch height) differences between the sexes (red symbols = females, blue symbols = males) of slender (squares) and Gaige's (triangles) anoles among one‐ and two‐species islands. Left: in the absence of interspecific competition, male slender anoles perch higher than females, although this pattern was not statistically significant on Island P. Right: In the presence of a competitor, niche overlap tends to increase between male and female slender anoles. Data are pooled across generations and symbols represent mean ± *SEM*. The asterisk denotes a significant difference.

Slender anoles and Gaige's anoles are likely competitors as they are similar in body size and are both generalist arthropod predators (Andrews, 1991; Köhler et al., 2012). These species also overlap in perch height, indicating that they may compete for space (confirmed by the coefficient estimates and standard errors for a mixed‐effects model; Figure 3; Table S4).

**FIGURE 4:**
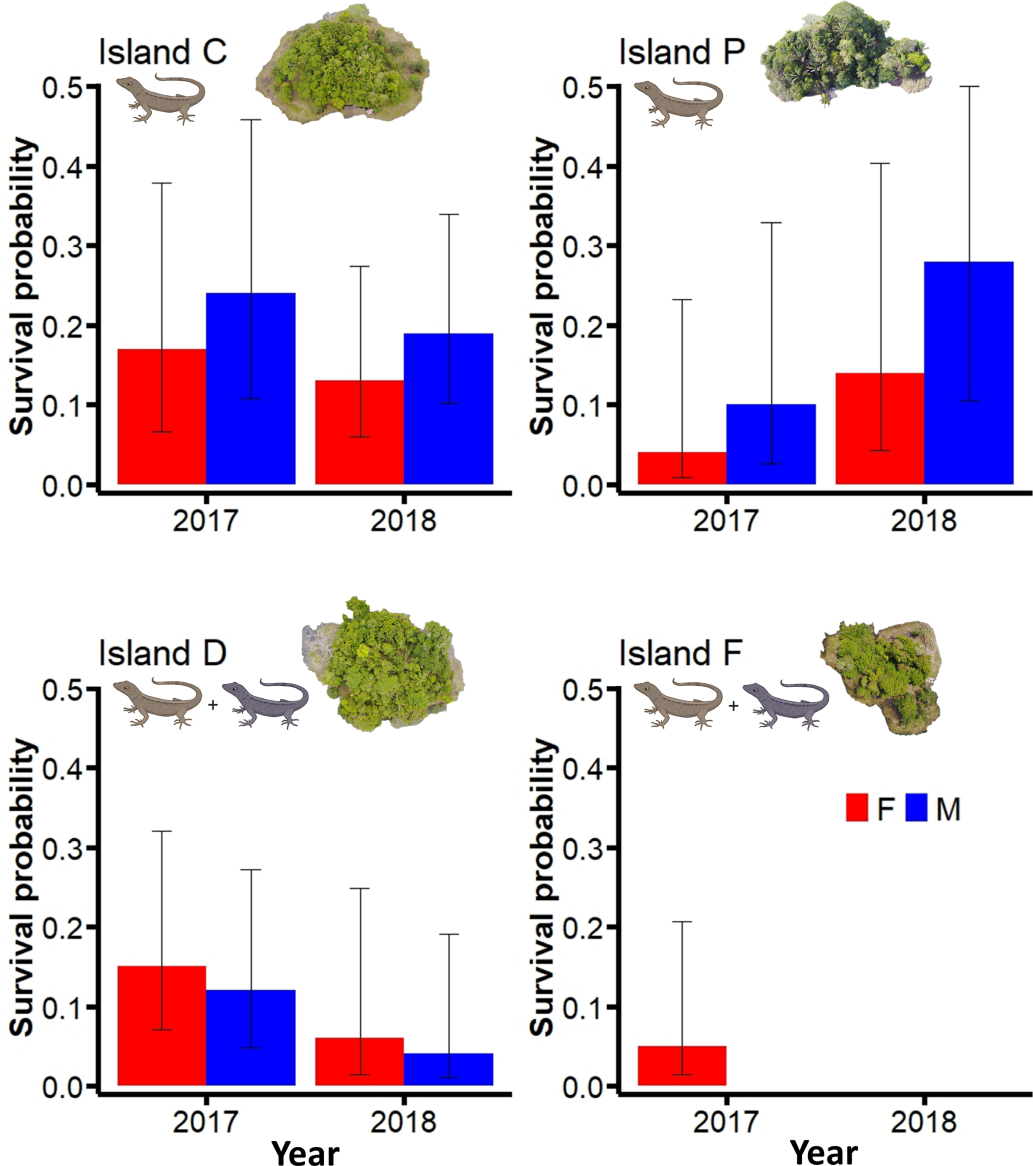
Differences in annual survival probability for males (blue bars) and females (red bars) among islands and years. Top row: males were more likely to survive on Islands C and P, which lacked a native competitor. Bottom row: Females were more likely to survive on Islands D and F which had a native competitor (Gaige's anole). Error bars represent upper and lower confidence limits. *Note*: the number of males on island P were so low that survival estimates were not possible.

Of the four islands to which we transplanted lizards in 2017, population size increased on the one‐species islands (Islands C and P) from the first to the second generations (credible intervals for population sizes between these years were entirely or mostly nonoverlapping for both islands; Table S3), followed by a decline in the third generation after the drought (Figure 2a). In contrast, we observed continuous population declines of slender anoles over the study period on the two‐species islands (credible intervals for population sizes between years were entirely nonoverlapping for both islands; Table S3), with near extinction by the third generation (Figure 2a,c). While slender anole survival rates were higher on the one‐species islands, population densities of slender anoles transplanted in 2017 were similar across all islands in 2018, irrespective of competitor presence (Figures 2c and 4).

### 3.3 Sex‐specific survival and habitat use

Among islands whereby differences in survival rate between the sexes could be analyzed (those founded in 2017), males had higher mean survival probabilities than females on one‐species islands, whereas females were more likely to survive on two‐species islands, across all years (Figure 4). However, the credible intervals for estimates of survival probability broadly overlapped between the sexes in all years (Table S5). Male slender anoles perch higher than females on the mainland (Logan et al., 2021), and this difference in perching behavior between the sexes was also apparent on our one‐species experimental islands (although the pattern was only statistically significant on Island C; confirmed by the coefficient estimates and standard errors from a mixed‐effects model; Figure 3; Table S4). Conversely, male and female slender anoles did not differ in perch height on the two‐species islands (Figure 3). The same mixed‐effects model revealed that there were no significant differences in perch height when comparing within sexes across one versus two‐species islands (Figure 3; Table S4).

## 4 DISCUSSION

Field‐experimental studies on the processes that facilitate establishment during biological invasion are rare. We translocated slender anole populations to islands in the Panama Canal and found that both a chance climate anomaly and the community structure of islands affected population dynamics across multiple generations. The populations that remained viable for at least three generations were those that had more time on the islands (two generations) prior to the drought, potentially allowing them to grow sufficiently or adapt to local conditions before the environment shifted. Populations were also more likely to remain viable in the absence of interspecific competition. Finally, we found limited evidence that males and females differed in their chances of survival depending on ecological context. Males appeared to survive longer than females in the absence of interspecific competition whereas females appeared to survive longer than males in the presence of interspecific competition, and this pattern held across years. Our results suggest that competition, climate stochasticity, and sex‐biased trait differences in the colonizing population may be important factors limiting establishment success in invasive species.

The final dry season of our study was drier than 93% of dry seasons over the previous 90 years according to data from the El Caro weather station on BCI, and the Panama Canal Association asserted that it was the most severe drought on record in central Panama (Fountain, 2019). It is plausible that this drought resulted in severe water stress on our transplanted populations, especially as the experimental islands were warmer than the mainland even in non‐drought years. Droughts can also have indirect effects by altering prey abundances and changing habit structure due to higher flora mortality (Walls et al., 2013). Studies have shown that precipitation levels influence reproductive output, habitat use, and population size in lizards (Andrews, 1991; Ryan et al., 2016; Stapley et al., 2015; Wang et al., 2016). Indeed, the four islands to which we translocated lizards only 4 months before the drought had effectively gone extinct by the beginning of the following wet season (less than a year later), even though these islands did not have a native competitor present. In contrast, the two one‐species islands that we translocated during the wet season of 2017—nearly 2 years and two lizard generations before the onset of the drought—were able to maintain much larger population sizes into the third generation. Because these islands were hotter (and therefore likely drier) than the ancestral (mainland) environment from which these lizards came, it is possible that these populations were able to adapt (through genetic change, plasticity, or both) to drought‐like conditions before exposure to the regional climate anomaly. Taken together, the growth trajectories of our experimental populations indicate that the timing of colonization relative to the timing of extreme weather events (or other environmental disturbances that shift local fitness optima) will be an important factor determining invasion success for some species.

The drought alone did not constrain establishment success. Instead, it interacted with the community context in which slender anoles found themselves after colonization. Two of the four islands to which we transplanted slender anoles in 2017 had resident populations of a congener, Gaige's anole. Slender anoles and Gaige's anoles likely compete with one another, as these species are similar in body size, are both generalist arthropod predators, and their habitat use (perch height) overlaps (Figure 3). These are conditions that are commonly expected to result in competition between species of anoles (Delaney & Warner, 2017; Dufour et al., 2020; Grether et al., 2009; Johnson et al., 2009; Vanhooydonck et al., 2005). Prior to the drought, slender anole population size and density declined most on the two‐species islands (Figure 2). After the drought, slender anole populations on two‐species islands further declined to near extinction, suggesting that competition had decreased population fitness beyond the threshold at which recovery from drought conditions may have been possible. These results support previous studies which argue that the presence of “enemies” in the colonized region can reduce the chance of successful invasion (Cox et al., 2020; Fey et al., 2019; Fey & Herren, 2014), while also highlighting the potential role of interactions between community structure and climatic variation.

While extrinsic features of the colonized environment such as community structure and climate may affect establishment success and thus the likelihood of invasion, endogenous aspects of the colonizing population may also play a role. For example, in sexually dimorphic species, especially those where the sexes occupy different ecological niches, competition and climate stochasticity are unlikely to impact males and females equally (Clutton‐Brock et al., 2002; Rankin & Kokko, 2007). We found that male slender anoles had higher mean survival rates on islands where native competitor species were absent while females had mean higher survival rates on islands with an endemic competitor, resulting in skewed population sex ratios almost immediately after colonization. While this pattern persisted for the length of our study, it is important to note that the credible intervals around our estimates of mean survival rate broadly overlapped between the sexes, and thus this result should be interpreted cautiously. Regardless, knowledge of the natural history of iguanian lizards suggests that males may have had reduced survival on two‐species islands because they tend to be the more territorial sex, spending more time actively defending a region of space against individuals of related species (Iglesias‐Carrasco et al., 2020). Previous studies suggest that males tend to have lower survival rates in the presence of interspecific competition due to increased energy invested into territory defense and display behavior (Galliard & Fitze, 2005; Seddon et al., 2013; Travers, 2015). Examination of perch height trends among islands showed increased niche overlap between male and female slender anoles on two‐species islands (smaller differences in means and overlapping standard errors; Figure 3), presumably because of competition for space with Gaige's anoles, and this could have further reduced survival of both sexes via increased intraspecific competition (Bolnick et al., 2010). Nevertheless, it is important to note that we did not find statistical differences in the perch height of either male or female slender anoles when comparing them between one and two‐species islands, suggesting that the effects of Gaige's anoles on slender anole habitat use were subtle and indicating the need for further research on interactions between the two species. In contrast to reduced survival of males on two‐species islands, males may have survived longer than females on one‐species islands, and this could be because the islands were warmer than the ancestral environment, and male slender anoles have slightly higher heat tolerance than females (Logan et al., 2021). In support of this hypothesis, the only individuals left on the four one‐species islands that were decimated by the drought were males. In the absence of interspecific competition, males may also be better at acquiring resources or evading island‐based predators. Our results imply that sex‐based trait differences in colonizing populations may play an important role in establishment success, and this should be especially apparent in sexually dimorphic species. Our results further indicate that differential survival of males and females in the aftermath of colonization may lead to faster population collapse than one would predict if sex‐based differences are not considered.

Due to human activity, the movement of alien species around the globe will continue to increase (Crozier & Dwyer, 2006; Pimentel et al., 2000; Urban, 2015), as will global temperatures and the frequency of extreme weather events (IPCC, 2021; McLaughlin et al., 2002). Thus, it is crucial that conservation biologists understand the factors that increase the likelihood that alien species will establish outside their native range. We found that in novel environments, stochastic climatic events interact with competition to determine the establishment success of an experimentally generated “alien species,” and these dynamics played out differently between the sexes. Although extreme weather events might be expected to weaken local communities and increase their vulnerability to invasion (McLaughlin et al., 2002), we found instead that climate anomalies can eliminate an invading population if the anomaly occurs before that population has had a chance to adjust to local conditions. Community context was equally important, as the presence of a native competitor interacted with climate to limit establishment success. Finally, females benefitted from interspecific competition but may have suffered under extreme weather, while the opposite was true for males, highlighting the ways in which invasion might play out differently between the sexes. Thus, accurate predictions of the likelihood of invasion will require an understanding of how features of the environment interact with endogenous aspects of the alien species to drive population dynamics.

## AUTHOR CONTRIBUTIONS

**Robert J. Knell:** Formal analysis (supporting); supervision (supporting); validation (supporting); visualization (supporting); writing – original draft (supporting); writing – review and editing (supporting). **Rachel S. McCrea:** Formal analysis (supporting); validation (supporting); writing – review and editing (supporting). **Lauren K. Neel:** Data curation (equal); investigation (equal); methodology (equal); project administration (equal); writing – original draft (supporting); writing – review and editing (supporting). **John David Curlis:** Data curation (equal); funding acquisition (equal); investigation (equal); methodology (equal); project administration (equal); writing – original draft (supporting); writing – review and editing (supporting). **Claire E. Williams:** Data curation (supporting); formal analysis (supporting); writing – original draft (supporting); writing – review and editing (supporting). **Albert K. Chung:** Data curation (equal); funding acquisition (equal); investigation (equal); methodology (equal); writing – original draft (supporting); writing – review and editing (supporting). **W. Owen McMillan:** Funding acquisition (equal); project administration (equal); resources (equal); supervision (supporting). **Trenton W. J. Garner:** Conceptualization (equal); supervision (supporting); writing – original draft (supporting); writing – review and editing (supporting). **Christian L. Cox:** Conceptualization (equal); data curation (equal); funding acquisition (equal); investigation (equal); methodology (equal); project administration (equal); supervision (supporting); writing – original draft (supporting); writing – review and editing (supporting). **Michael L. Logan:** Conceptualization (equal); data curation (equal); funding acquisition (equal); investigation (equal); methodology (equal); project administration (equal); supervision (lead); writing – original draft (supporting); writing – review and editing (supporting). **Daniel J. Nicholson:** Conceptualization (equal); data curation (equal); formal analysis (lead); funding acquisition (equal); investigation (equal); methodology (equal); project administration (equal); visualization (lead); writing – original draft (lead); writing – review and editing (lead).

## FUNDING INFORMATION

This project was supported by a NERC studentship (NE/L002485/1) a Smithsonian Pre‐Doctoral Fellowship, and a Smithsonian Tropical Research Institute Bridge Fund Grant awarded to DJN, a National Science Foundation grant (DEB‐2024157), and Smithsonian Institution Biodiversity Genomics Postdoctoral and Smithsonian Tropical Research Institute Earl S. Tupper Postdoctoral Fellowships awarded to MLL. Additional funding included an American Museum of Natural History Theodore Roosevelt Memorial Research grant, Georgia Southern University Graduate Student Professional Development funds, and Smithsonian Tropical Research Institute short‐term fellowships awarded to AKC and JDC. RSM was funded by the Engineering and Physical Sciences Research Council (EP/S020470/1).

## CONFLICT OF INTEREST

All authors declare no competing interests.

## Supporting information


Appendix S1

## Data Availability

Data contributed to this paper are owned by the authors and full permission for commercial use is given. Data can be found in a dryad repository: https://doi.org/10.5061/dryad.dfn2z3546 and data will be made available after publication.
