# Supplementary material for: Climate anomalies and competition reduce establishment success during island colonization
Source: Ecol Evol. 2022 Oct 8;12(10):e9402. doi: 10.1002/ece3.9402 (PMC9547383; doi:10.1002/ece3.9402)
Supplement: Supplementary file 1 — Appendix S1 [file ECE3-12-e9402-s001.docx]

**Supplemental Information:**

| Table S1: AIC values and models for population size and sex-based survival estimates. | | | | |  |
| --- | --- | --- | --- | --- | --- |
| **Model** | **Site** | **Parameters** | **Number of parameters** | **AICc** |  |
|  |  |  |  |  |  |
| Population size | Island C | S(week)p(year) | 9 | 603.631 |  |
|  |  | S(.)p(week) | 18 | 749.302 |  |
|  |  | S(week)p(week) | 19 | 751.354 |  |
|  |  | S(.)p(.) | 6 | 855.943 |  |
|  |  | S(week)p(.) | 7 | 857.848 |  |
|  | Island D | S(week)p(year) | 9 | -2.833 |  |
|  |  | S(.)p(week) | 18 | 109.548 |  |
|  |  | S(week)p(week) | 19 | 110.478 |  |
|  |  | S(.)p(.) | 6 | 170.753 |  |
|  |  | S(week)p(.) | 7 | 171.281 |  |
|  | Island F | S(week)p(year) | 9 | -63.838 |  |
|  |  | S(.)p(week) | 18 | 8.559 |  |
|  |  | S(week)p(week) | 19 | 10.588 |  |
|  |  | S(.)p(.) | 6 | 25.704 |  |
|  |  | S(week)p(.) | 7 | 27.249 |  |
|  | Island P | S(week)p(year) | 9 | 19.161 |  |
|  |  | S(week)p(week) | 19 | 138.969 |  |
|  |  | S(.)p(week) | 18 | 141.518 |  |
|  |  | S(week)p(.) | 7 | 231.291 |  |
|  |  | S(.)p(.) | 6 | 234.511 |  |
| Sex-based survival | Island C | S(week + sex)p(year) | 10 | 880.859 |  |
|  |  | S(. + sex)p(week) | 19 | 1021.489 |  |
|  |  | S(week + sex)p(week) | 20 | 1023.6042 |  |
|  |  | S(. + sex)p(.) | 7 | 1131.7019 |  |
|  |  | S(week + sex)p(.) | 8 | 1133.665 |  |
|  | Island D | S(week + sex)p(year) | 10 | 148.649 |  |
|  |  | S(. + sex)p(week) | 19 | 253.883 |  |
|  |  | S(year + sex)p(week) | 20 | 254.955 |  |
|  |  | S(. + sex)p(.) | 7 | 314.688 |  |
|  |  | S(week + sex)p(.) | 8 | 315.351 |  |
|  | Island F | S(week + sex)p(year) | 10 | 42.543 |  |
|  |  | S(. + sex)p(year) | 19 | 116.018 |  |
|  |  | S(week + sex)p(week) | 20 | 117.835 |  |
|  |  | S(. + sex)p(.) | 7 | 132.221 |  |
|  |  | S(week + sex)p(.) | 8 | 133.553 |  |
|  | Island P | S(week + sex)p(year) | 10 | 187.863 |  |
|  |  | S(week + sex)p(week) | 20 | 305.527 |  |
|  |  | S(. + sex)p(week) | 19 | 308.375 |  |
|  |  | S(week + sex)p(.) | 8 | 395.457 |  |
|  |  | S(. + sex)p(.) | 7 | 398.817 |  |

S(week) = weekly variation in apparent survival; S(.) = no variation; p(year) = yearly variation in capture probability; p(week) = weekly variation in capture probability; p(.) = no variation. Years represent primary occasions (field seasons) and weekly represents secondary occasions (each week of sampling during the field season).

| Table S2: Description of mixed effect models for BCI temperature data. | | | |  |
| --- | --- | --- | --- | --- |
| **Season** | **β ± *SE* ^(a)^** | **95% CIs** | **Random effect** |  |
|  |  |  |  |  |
| Dry Season 2017 | 25.735 ± 0.070 | 25.598, 25.872 | 0.852 |  |
| Dry Season 2018 | 0.109 ± 0.099 | -0.084, 0.303 |  |  |
| Dry Season 2019 | 1.045 ± 0.099 | 0.851, 1.239 |  |  |
| ^(a)^ Coefficient estimate and the corresponding standard error | | | |  |

| Table S3: Population estimates and credible intervals for all islands and years | | | | |  |
| --- | --- | --- | --- | --- | --- |
| **Island** | **Year** | **Population size** | **Lower CI** | **Upper CI** |  |
|  |  |  |  |  |  |
| Island C | 2017 | 70 | 70 | 70 |  |
|  | 2018 | 169 | 115 | 328 |  |
|  | 2019 | 59 | 51 | 87 |  |
| Island D | 2017 | 69 | 69 | 69 |  |
|  | 2018 | 43 | 38 | 67 |  |
|  | 2019 | 8 | 8 | 8 |  |
| Island F | 2017 | 70 | 70 | 70 |  |
|  | 2018 | 13 | 13 | 12 |  |
|  | 2019 | 2 | 2 | 2 |  |
| Island P | 2017 | 70 | 70 | 70 |  |
|  | 2018 | 76 | 40 | 246 |  |
|  | 2019 | 31 | 26 | 62 |  |

| Table S4: Description of mixed effect models for habitat use. | | | | |  |
| --- | --- | --- | --- | --- | --- |
| **Mixed effect model** | **Season/Site/Sex/Species** | **β ± *SE* ^(a)^** | **95% CIs** | **Random effect** |  |
|  |  |  |  |  |  |
| Perch height between species | Island D - *A.apletophallus* | 4.137 ± 0.077 | 3.985, 4.288 | 0.48 |  |
|  | Island D - *A. gaigei* | 0.140 ± 0.119 | -0.091, 0.372 |  |  |
|  | Island F - *A. apletophallus* | -0.140 ± 0.122 | -0.554, 0.058 |  |  |
|  | Island F - *A. gaigei* | 0.014 ± 0.193 | -0.362, 0.392 |  |  |
| Perch height between sex | Island C - Female | 3.857 ± 0.061 | 3.737, 3.976 | 0.016 |  |
|  | Island C - Male | 0.384 ± 0.083 | 0.222, 0.547 |  |  |
|  | Island D - Female | 0.173 ± 0.137 | -0.092, 0.439 |  |  |
|  | Island D - Male | -0.209 ± 0.178 | -0.556, -0.136 |  |  |
|  | Island F - Female | 0.083 ± 0.152 | -0.213, 0.379 |  |  |
|  | Island F - Male | -0.279 ± 0.035 | -0.681, 0.122 |  |  |
|  | Island P - Female | 0.151 ± 0.114 | -0.069, 0.373 |  |  |
|  | Island P - Male | -0.031 ± 0.158 | -0.340, 0.275 |  |  |
| ^(a)^ Coefficient estimate and the corresponding standard error | | | | |  |

| Table S5: Survival estimates and credible intervals for all islands, sex, and years | | | | | |  |
| --- | --- | --- | --- | --- | --- | --- |
| **Island** | **Year** | **Sex** | **Survival** | **Lower CI** | **Upper CI** |  |
|  |  |  |  |  |  |  |
| Island C | 2017 - 2018 | Female | 0.17 | 0.066 | 0.379 |  |
|  |  | Male | 0.24 | 0.1085 | 0.4588 |  |
|  | 2018 - 2019 | Female | 0.13 | 0.06 | 0.274 |  |
|  |  | Male | 0.19 | 0.1015 | 0.3392 |  |
| Island D | 2017 - 2018 | Female | 0.15 | 0.0702 | 0.3201 |  |
|  |  | Male | 0.12 | 0.0483 | 0.2714 |  |
|  | 2018 - 2019 | Female | 0.06 | 0.014 | 0.249 |  |
|  |  | Male | 0.04 | 0.0104 | 0.1914 |  |
| Island F | 2017 - 2018 | Female | 0.05 | 0.0147 | 0.207 |  |
|  |  | Male | 0 | 0 | 0 |  |
|  | 2018 - 2019 | Female | 0 | 0 | 0 |  |
|  |  | Male | 0 | 0 | 0 |  |
| Island P | 2017 - 2018 | Female | 0.04 | 0.0086 | 0.233 |  |
|  |  | Male | 0.1 | 0.0261 | 0.329 |  |
|  | 2018 - 2019 | Female | 0.14 | 0.0432 | 0.4043 |  |
|  |  | Male | 0.28 | 0.1057 | 0.5 |  |
